# WYC-209 inhibited GC malignant progression by down-regulating WNT4 through RARα

**DOI:** 10.1080/15384047.2023.2299288

**Published:** 2024-01-04

**Authors:** Zhenyuan Qian, Wenfa Lin, Xufan Cai, Jianzhang Wu, Kun Ke, Zaiyuan Ye, Fang Wu

**Affiliations:** a General Surgery, Cancer Center, Department of Gastrointestinal and Pancreatic Surgery, Zhejiang Provincial People’s Hospital, Affiliated People’s Hospital, Hangzhou Medical College, Hangzhou, Zhejiang, China; b School of Medicine, Zhejiang Chinese Medical University, Hangzhou, Zhejiang, China

**Keywords:** GC, WYC-209, synthetic retinoid, WNT4, RARα

## Abstract

Gastric cancer (GC) has been a major health burden all over the world but there are fewer promising chemotherapeutic drugs due to its multidrug resistance. It has been reported that WYC-209 suppresses the growth and metastasis of tumor-repopulating cells but the effect on GC was not explored. MTT, colony formation, and transwell assays were performed to examine the effects of WYC-209 on the proliferation, colony growth, and mobility of GC cells. Western blotting and qRT-PCR were used to detect the expression of proteins and mRNA. RNA-seq and enrichment analyses were conducted for the differentially expressed genes and enriched biological processes and pathways. The rescue experiments were carried out for further validation. Besides, we constructed xenograft model to confirm the effect of WYC-209 in vivo. The dual-luciferase reporter and Chromatin immunoprecipitation were implemented to confirm the underlying mechanism. WYC-209 exerted excellent anti-cancer effects both in vitro and in vivo. Based on RNA-seq and enrichment analyses, we found that Wnt family member 4 (WNT4) was significantly down-regulated. More importantly, WNT4 overexpression breached the inhibitory effect of WYC-209 on GC progression. Mechanically, WYC-209 significantly promoted the binding between retinoic acid receptor α (RARα) and WNT4 promoter. WYC-209 exerts anti-tumor effects in GC by down-regulating the expression of WNT4 via RARα.

## Introduction

Known as the second leading reason of cancer death following lung cancer,^1^ gastric cancer (GC) has a miserable prognosis which can be exemplified by the 5-y survival rate below 20%.^2^ Nowadays, the incidence of GC has declined apparently due to the increased emphasis on a healthy diet and improved hygiene standards.^3^ But worryingly, the therapy of GC remains non-guaranteed, for there are fewer promising chemotherapeutic drugs.^4^ So the exploration of effective anti-tumor therapy or drugs for GC is still an imminent endeavor.

Retinoids are a collection of vitamin A and its derivatives (natural and synthetic analogues). Accumulating studies have proved that retinoids are engaged in multiple biological processes, including cell proliferation, differentiation, embryogenesis, and metabolism.^5^ In the last two decades, the application of retinoids in anti-cancer therapy has progressed a lot. For example, the promising anti-tumor efficacy of all-trans retinoic acid (ATRA), 13-cis-RA, and 9-cis-RA have been widely reported *in vivo* .^6–8^ The function of retinoids is addressed through hormone receptors (NRs), including the retinoic acid receptors (RARs) and the retinoid X receptors (RXRs).^9^

Although retinoids were promising for normal or malignant diseases, the low water solubility based on their structure and the high toxicity at high doses were an obstacle to developing new drugs.^10,11^ Fortunately, synthetic retinoids have introduced an amide linkage that reduces lipophilicity and this is helpful for their affinity for RAR, especially RARα.^12^ Moreover, it seems that synthetic retinoids exert stronger anti-cancer activity with fewer adverse events compared with natural retinoids, for example, ST1926 (also named adarotene), has been reported to have better effects on the cell growth MCF-7 and MDA-MB-231 cells compared with that of ATRA.^13^ WYC-209 studied in this work, is a novel synthetic retinoid analogue designed from the class III retinoid Tazarotene. It has been proved that WYC-209 has negligible toxicity in non-cancerous cells but inspiringly inhibited the proliferation of tumor-repopulating cells (TRCs),^14^ which is potentially tumorigenic and indispensable in tumor development.^15^ Given that WYC-209 exhibited inspiring capacity in antitumor therapy, we tried to investigate the effect of WYC-209 on GC and wondered about the mechanisms it works.

## Results

### *WYC-209 exerted prospective anti-cancer effects* in vitro

**Figure 1.**
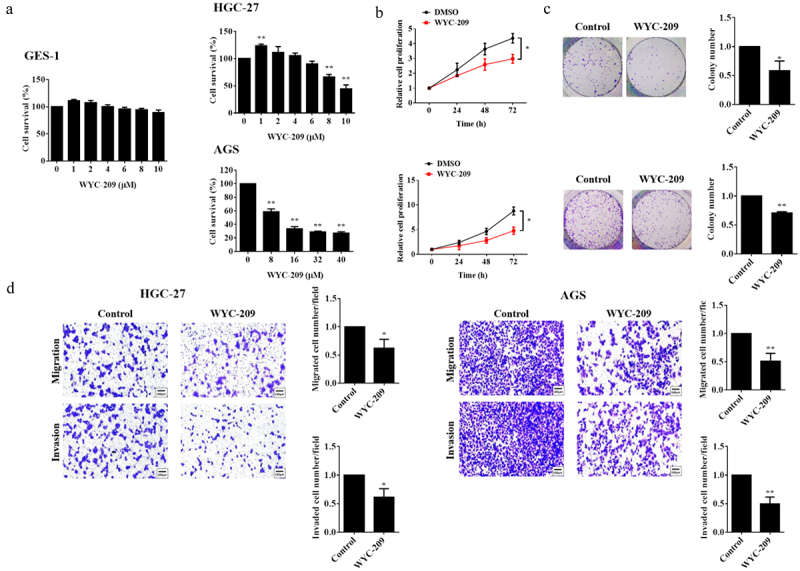
WYC-209 inhibited the cell survival, proliferation, colony growth, and motility of GC cells.

It has been reported that retinoids have already been utilized as prospective cancer therapeutic agents because of the reported anti-proliferative, pro-apoptotic as well as antioxidant roles.^16^ So in this study, we wondered about the effects of WYC-209, a novel synthetic retinoid analogue, on GC. Preliminarily, we examined the toxicity of the drug on non-cancerous cells (GES-1) and two human gastric cancer cell lines. As shown in Figure 1a, 8 μM of WYC-209 treatment significantly inhibited cell survival, and its inhibitory effect was positively related to concentration (**P < .01), yet it didn’t affect the cell survival of GES-1. Based on this result, 8 μM of WYC-209 treatment was applied for the following experiments. Furthermore, WYC-209 demonstrated its provocative suppression of cell proliferation, colony-forming ability, migration as well as invasion (Figure 1b–d, **P < .01, *P < .05).

(a) The cell survival of GES-1 under 0, 1, 2, 4, 6, 8, 10 μM of WYC-209 treatment, HGC-27 under 0, 1, 2, 4, 6, 8, 10 μM of WYC-209 treatment and AGS cells under 0, 8, 16, 32, 40 μM of WYC-209 treatment was examined by MTT assay. The histogram represents the quantified results, as below. (b) The cell proliferation of HGC-27 and AGS cells under 8 μM of WYC-209 treatment for 24 h was examined by MTT assay. (c) The colony growth ability of HGC-27 and AGS cells under 8 μM of WYC-209 treatment was examined by colony formation assay. (d) The migration and invasion capacity of HGC-27 and AGS cells under 8 μM of WYC-209 treatment for 24 h was examined by transwell experiments. Results were analyzed based on three repeatable experiments.**P < .01, *p < .05.

**Figure 2.**
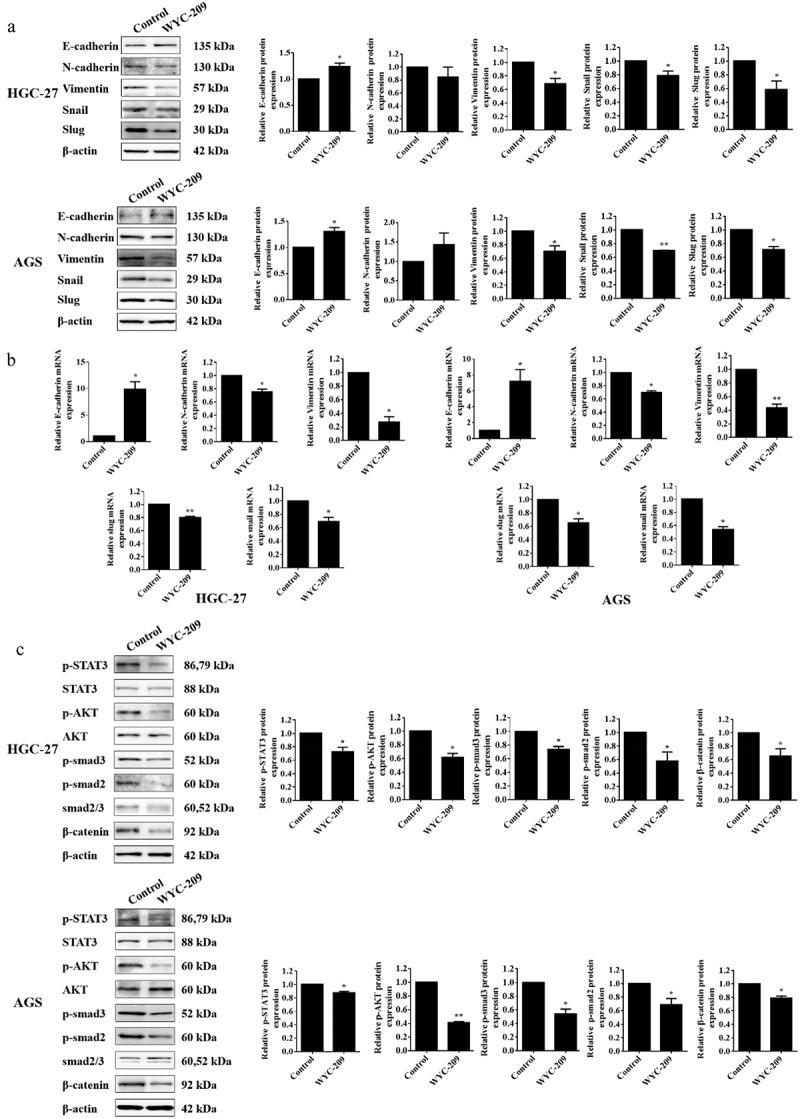
WYC-209 suppressed EMT and tumor progression.

Furthermore, we found that WYC-209 treatment significantly up-regulated the expression of epithelial biomarker E-cadherin and down-regulated the expression of mesenchymal biomarkers N-cadherin and vimentin. Besides, the expression levels of the Snail and Slug, were also distinctly subdued due to WYC-209, revealing that this agent played an indispensable role in inhibiting EMT (epithelial-mesenchymal transition) process (Figure 2a, **P < .01, *P < .05), which was further illustrated by the results from subsequent qRT-PCR experiments (Figure 2b, **P < .01, *P < .05).

(a) The EMT-related protein levels expressed in HGC-27 and AGS cells were detected by WB assay after 8 μM of WYC-209 treatment for 24 h. The histogram represents the quantified results, as below. (b) The EMT-related mRNA levels expressed in HGC-27 and AGS cells were detected by qRT-PCR after 8 μM of WYC-209 treatment for 24 h. (c) The phosphorylation of STAT3, AKT, smad2, smad3, and β-catenin expression levels in HGC-27 and AGS cells with 8 μM of WYC-209 treated for 24 h were tested by WB assay. Results were analyzed based on three repeatable experiments. **P < .01, *p < .05.

Besides, we examined the phosphorylation of STAT3, AKT, smad2, and smad3 as well as β-catenin, which were always activated in tumorigenesis and progression.^17–20^ As shown in Figure 2c, after WYC-209 treatment, the expression levels of p-STAT3, p-AKT, p-smad2, p-smad3, and β-catenin were all impeded significantly (**P < .01, *P < .05), further pointed out that WYC-209 could suppress tumor progression and was potential for antitumor therapy.

### WYC-209 induced the down-regulation of WNT4 in GC

**Figure 3.**
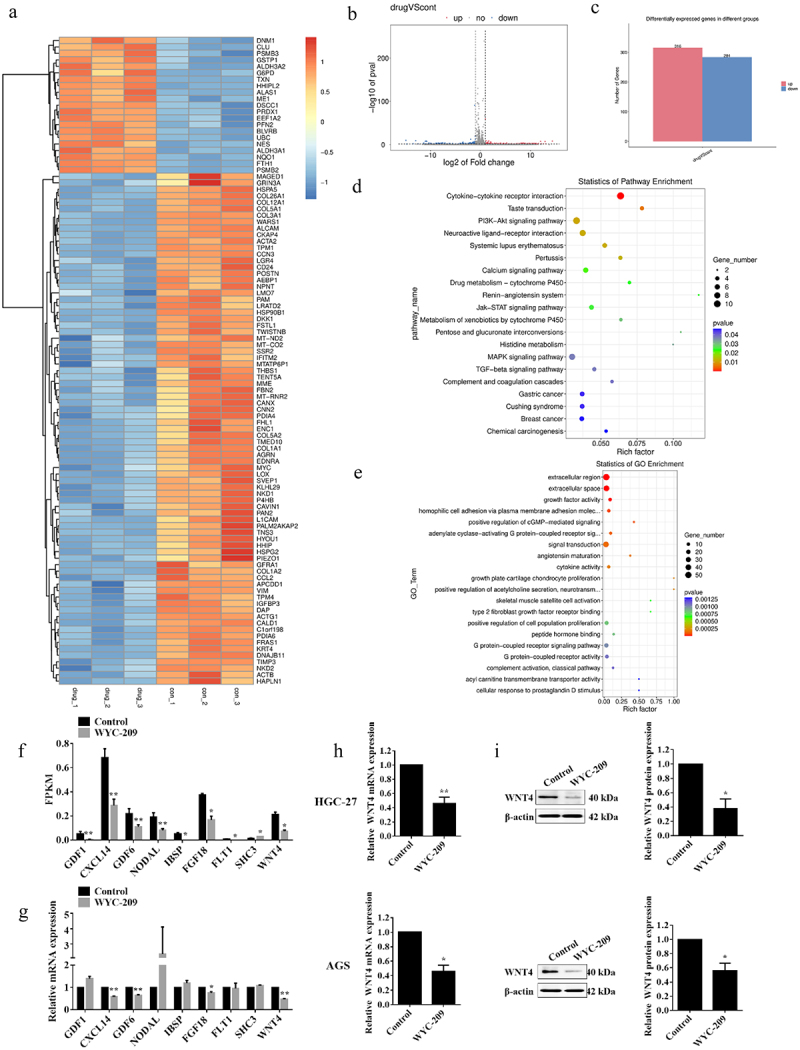
WYC-209 induced the heterotypic down-regulation of WNT4.

To further investigate the underlying mechanism of WYC-209 functions, we performed RNA-seq in HGC-27 cells with or without WYC-209 treated for 24 h to detect DEGs. The sequencing outcomes told us that the agent led to 316 up-regulated and 284 down-regulated genes (Figure 3a–c). Furthermore, enrichment analyses underscored the PI3K-Akt, Jak-STAT pathway, and the biological processes like growth factor activity, signal transduction, and the regulation of cell proliferation (Figure 3d, e). Subsequently, according to the fold change of DEGs and enrichment analysis results, we selected 9 genes for the Fragments per Kilobase Million (FPKM) analysis and qRT-PCR validation (Figure 3f, h, **P < .01, *P < .05). Interestingly, the results revealed that the WNT4 mRNA level was statistically decreased. And the following qRT-PCR and WB experiments further supported that at both mRNA and protein levels (Figure 3h, i, **P < .01, *P < .05). These data were meaningful for it has been reported that WNT4 was engaged in multiple human diseases and was always up-regulated in tumor tissues.^21^

(a, b&c) The total RNA of HGC-27 cells was extracted after 8 μM of WYC-209 treatment for 24 h, and then RNA-seq was performed to analyze differentially expressed genes (DEGs). The heatmap and the volcano plot were generated by the R package. (d&e) KEGG and GO analysis were carried out for the enriched biological processes and pathways. (f&g) FPKM and qRT-PCR validation of DEGs in HGC-27 cells based on RNA-seq results. (h) The qRT-PCR validation of WNT4 expression level in HGC-27 and AGS cells after 8 μM of WYC-209 treatment for 24 h. The histogram represents the quantified results, as below. (i) The WB validation of WNT4 expression level in HGC-27 and AGS cells after 8 μM of WYC-209 treatment for 24 h. Results were analyzed based on three repeatable experiments. **P < .01, *p < .05.

### Overexpressing WNT4 breached the inhibitory effect of WYC-209 on GC progression

**Figure 4.**
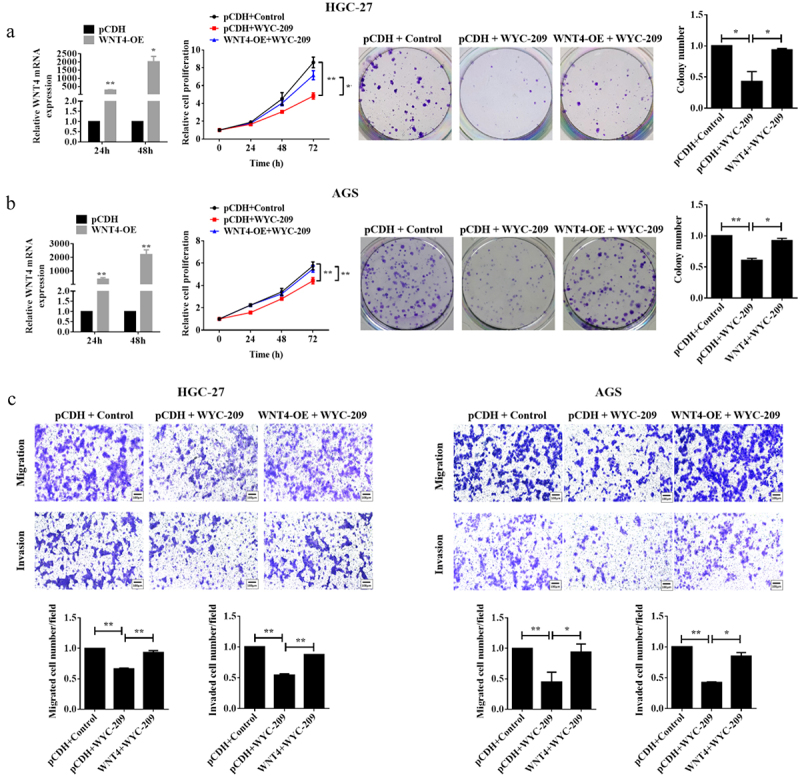
Overexpressing WNT4 breached the inhibitory effect of WYC-209 on cell proliferation, colony-forming, and motility.

**Figure 5.**
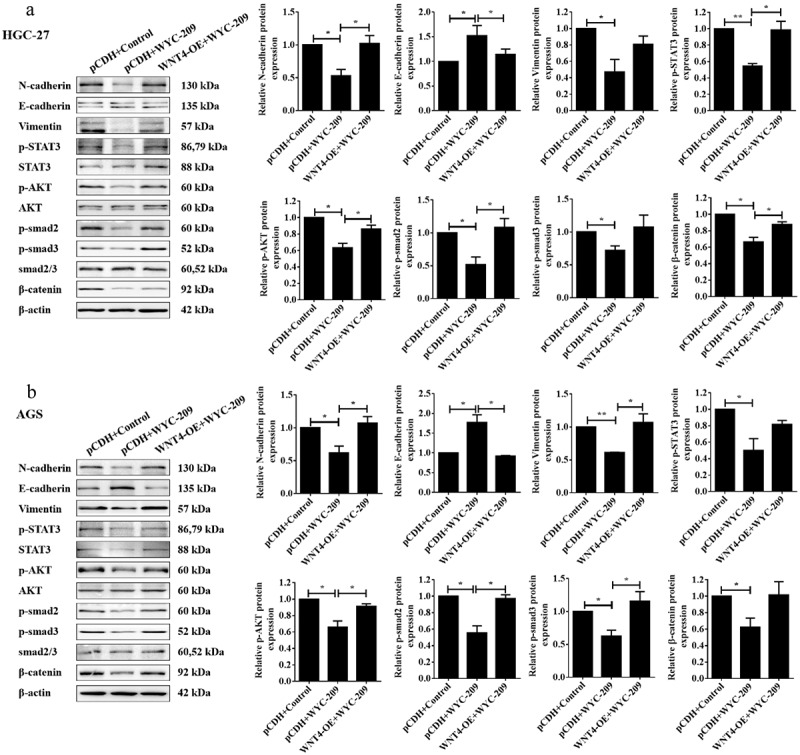
EMT and tumor progression inhibited by WYC-209 were abrogated by overexpressing WNT4.

According to the result that WYC-209 induced a significant down-regulation of WNT4 in GC, we next constructed rescue experiments for further validation. As shown in Figure 4a, b, WNT4-overexpressed GC cell lines were constructed and qRT-PCR was utilized to examine the transfection efficiency. The following MTT and colony formation assays pointed out that the overexpression of WNT4 countervailed the cell proliferation and colony-forming capacity impaired by WYC-209 (**P < .01, *P < .05). Additionally, the WYC-209-decreased cell mobility of HGC-27 and AGS cells was also reversed due to WNT4 overexpression (Figure 4c, *P < .05). More importantly, WNT4 overexpression caused significant reversal in the WYC-209-suppressed tumor progression because, after WNT4 overexpression, the expression of E-cadherin was decreased while the expression of N-cadherin and Vimentin were increased, while STAT3, AKT, smad2/3 as well as β-catenin were re-activated (Figure 5a, b, **P < .01, *P < .05). In aggregate, we could probably conclude that the inhibitory effect of WYC-209 on GC cells may be accomplished by down-regulating WNT4.

(a&b) To investigate the role of WNT4, the WNT4 overexpressed HGC27 and AGS cells were constructed. After qRT-PCR validation of WNT4 expression level, 8μM of WYC-209 was used to treat HGC27 and AGS cells with WNT4 overexpressed or not for 24h. Then MTT, colony formation, and (c) transwell assay were performed to detect the cell proliferation, colony-forming, migration, and invasion abilities. The histogram represents the quantified results. Results were analyzed based on three repeatable experiments. **P<.01, *p<.05.

(A&B) 8 μM of WYC-209 was used to treat HGC27 and AGS cells with WNT4 overexpressed or not for 24 h, then WB was carried out to examine the EMT-related proteins levels, the phosphorylation of STAT3, AKT, smad2, smad3 and β-catenin expression level in HGC-27 and AGS cells. The histogram represents the quantified results. Results were analyzed based on three repeatable experiments.**P < .01, *p < .05.

### WYC-209 inhibited GC progression by down-regulating WNT4 via RARα

**Figure 6.**
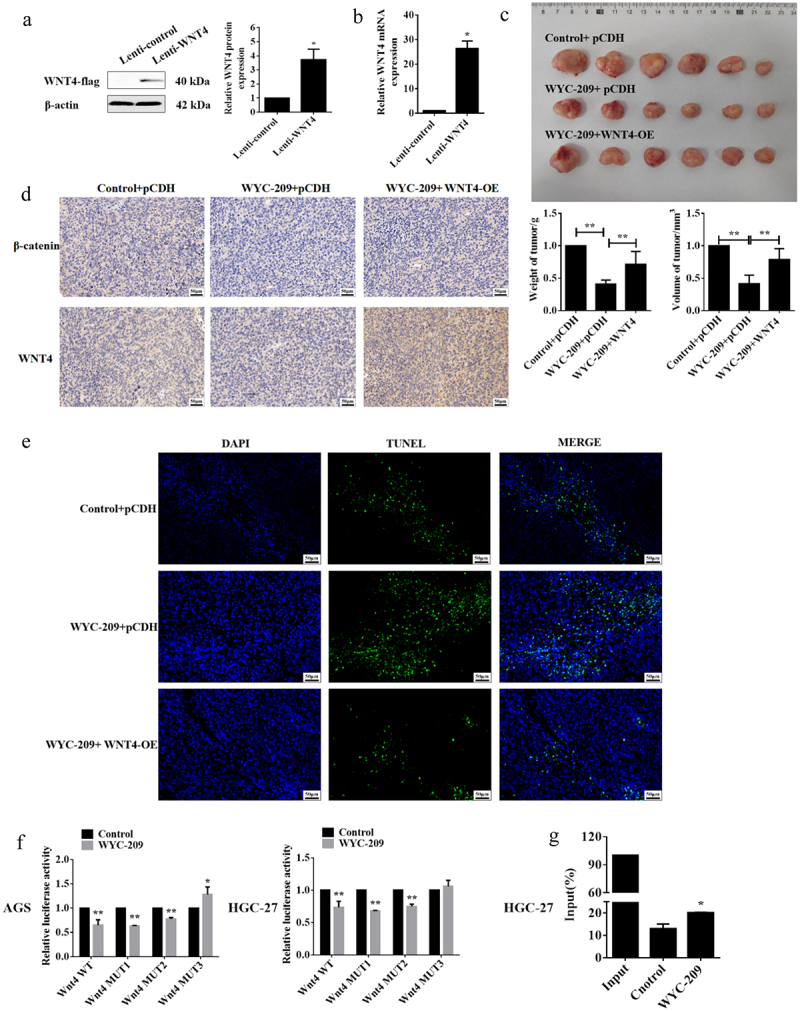
WYC-209 inhibited GC progression by down-regulating WNT4 via RARα.

Earlier we preliminarily confirmed the anti-cancer role of WYC-209 on GC cells and this function may be addressed by suppressing WNT4 expression. So for further investigation, xenograft models were constructed to validate the effect of WYC-209 *in vivo*. The WNT4 stably overexpressed HGC-27 cell line was generated using lentivirus and after WB and qRT-PCR validation for transfection efficiency (Figure 6a, b, *P < .05), the xenograft tumor models were constructed. Unsurprisingly, WYC-209 treatment indeed attenuated the weight and volume of the xenograft tumors, which could be recovered by overexpressing WNT4 (Figure 6c, **P < .01). And, the WYC-209-inhibited WNT4 and β-catenin protein levels expressed in the xenograft tumor tissues were reversed by WNT4 overexpression (Figure 6d), besides, the facilitated cell apoptosis induced by WYC-209 was also abrogated (Figure 6e). Collectively, we can conclude that WYC-209 played an anti-cancer part in GC both *in vitro* and *in vivo* by down-regulating WNT4, at least partially. Last but not least, dual-luciferase reporter and ChIP experiments were implemented for a more detailed mechanism. Primarily, we predicted the binding sites on the WNT4 promoter to RARα, a retinoic acid receptor, by the JASPAR website, and three probably binding sites were exhibited. The following dual-luciferase reporter pointed out that only the mutation of site 3 recovered the luciferase activity both in AGS and HGC-27 cells after WYC-209 treatment (Figure 6f, **P < .01, *P < .05), indicating that WNT4 bond to RARα at site 3, which was further proved by the subsequent ChIP experiment (Figure 6g, *P < .05). In aggregate, WYC-209 may down-regulate the expression of WNT4 via RARα, a retinoic acid receptor, and then decelerate GC progression.

## Discussion

Retinoids, including vitamin A and related derivatives, have been proven to own the anti-differentiation and pro-apoptosis capacity and thus can be utilized for cancer therapy,^22^ but the bleak water solubility and breakdown during intravenous administration greatly limit their efficacy and impede the application.^23^ Provocatively, synthetic retinoids are superior to endogenous retinoids, because their structures are more chemically stable.^13,24^ Besides, they always function through a specific RAR that results in minor side effects.^25^ For example, the synthetic retinoid ST1926 showed an inhibitory effect on the proliferation of MDA-MB-231 and MCF7 cells by attenuating the Wnt/β-catenin signaling pathway, more inspiringly, this inhibition was superior compared with that of ATRA.^13^ And bexarotene (LGD1069, Targretin) inhibited considerably non-small cell lung cancer when combined with erlotinib.^26^

In our study, we mainly concentrated on WYC-209, the new synthetic retinoid analogue, which has been reported to have an inhibitory effect on the proliferation of TRCs, including breast cancer, lung cancer, and so on. And importantly, its inhibitory effect on the growth of TRCs did not disappear after the drug washout.^14^ Furthermore, WYC-209 effectively suppressed multiple tumorigenic features and drug-resistance of cancer stem cells (CSCs) *in vitro*, and attenuated the tumor growth *in vivo*, with feeble side effects compared with sorafenib and acyclic retinoid.^27^ Accordingly, we investigated the effect of WYC-209 on GC and surprisingly, this novel retinoid analogue evidently inhibited the survival of HGC-27 and AGS cells, with a positive correlation to concentration. But notably, 1 μM of WYC-209 increased the survival of HGC-27, we guessed that this phenomenon might be related to the reversible functions of some small molecules because of their bleakly immunogenic, more permeable characteristics so that their functions can be tuned by regulating concentrations.^28^ More importantly, 8 μM WYC-209 suppressed the cell proliferation, colony-forming capacity, migration, and invasion of both HGC-27 and AGS cells, mitigated the EMT progression and the activation of classical oncogenic signaling pathways, such as STAT3, PI3K/AKT, TGF-β/smad2/3, and WNT/β-catenin. RNA-seq and enrichment analysis also underscored that the DEGs were enriched in the PI3K-Akt, Jak-STAT signaling pathway, and biological processes like growth factor activity, signal transduction, and the regulation of cell proliferation. Besides, we observed a significant down-regulation of WNT4 mRNA expression, so we wondered about the role of WNT4 in the WYC-209-induced effect on GC. By further qRT-PCR and WB validation, we confirmed that the expression of WNT4 protein and mRNA both declined significantly after WYC-209 treatment in HGC-27 and AGS cells. It has been proved that WNT4 is usually over-expressed in multiple tumor tissues, like breast cancer,^29^ colorectal cancer,^30^ and so on, and this contributes to cancer cell differentiation and tumor progression by activating β-catenin or β-catenin-independent pathways.^31^ And the activation of the WNT4/β-catenin pathway contributes to EMT progression and then induces enhanced cell motility,^30^ which further elaborated our results that WYC-209 down-regulated the WNT4 expression, then influenced the expression of β-catenin through WNT4/β-catenin pathway, as such, inhibited EMT process. This conclusion was further indicated because of the recovered WYC-209-induced suppression of GC progression both *in vitro* and *in vivo* after WNT4 overexpression treatment.

As mentioned, retinoids play their anti-cancer role via binding RAR. There are two subfamilies of receptors, one is RARs, binding naturally occurring retinoids and another is retinoid X receptors (RXRs). RARs own three isoforms: RARα, RARβ, and RARγ, respectively.^32^ Of note, Different retinoids may have different affinities to RARs and RXRs. It has been proved that ATRA binds RARs with better affinity,^33^ yet 9-cis retinoic acid can activate both RARs and RXRs.^34^ For WYC-209, researchers have indicated that the treatment of WYC-209 led to hyper-expression of RARα mRNA, but RARα-specific siRNAs breached the inhibitory effects of WYC-209 on TRCs, meaning that WYC-209 acts as an agonist of RARα.^27^ On this basis, we explored further mechanisms by which WYC-209 works. Interestingly enough, we found that there are three probably binding sites of RARα on the WNT4 promoter, but only the mutation of site 3 recovered the luciferase activity both in AGS and HGC-27 cells after WYC-209 treatment, indicating that RARα could mediate the transcription of WNT4 by binding to WNT4 promoter at site 3 and this could be further proved by ChIP experiments. Therefore, we can conclude that WYC-209 inhibited GC malignant progression by down-regulating WNT4 through RARα.

## Materials and methods

### Cells

In this study, the normal human gastric epithelial cell line GES-1 (CL-0563) was purchased from Procell Bio (Wuhan, China), and the human gastric cancer cell lines HGC-27 (Procell Bio, CL-0107) and AGS (Procell Bio, CL-0022) were selected and cultivated in DMEM (Gibco, Waltham, MA, USA, 02-5062EJ) which containing 10% fetal bovine serum (FBS, Gibco, Waltham, MA, USA, 10099141C) at 37°C with 5% CO_2_ supplied in an incubator (Thermofisher, Waltham, MA, NO. 311).

### Cell proliferation analysis

At first, for dose screening, the cell survival of HGC-27 under 0, 1, 2, 4, 6, 8, 10 μM of WYC-209 treatment and AGS cells under 0, 8, 16, 32, 40 μM of WYC-209 treatment for 24 h was examined by MTT assay. In short, MTT (Sigma-Aldrich, St. Louis, MO, USA, M2128) was dissolved in PBS at 1 mg/ml and added to each well (50 μl per well). After 3 h of reaction, 150 μl of dimethyl sulfoxide (DMSO) was added to dissolve the formed crystals. Finally, the OD value at 570 nm was determined using the microplate reader (Molecular Device, California, USA). Subsequently, HGC-27 and AGS cells with or without 8 μM of WYC-209 treated were allowed to attach at a density of 3000 cells/well in 96-well plates. Cells cultured for 24, 48, 72, and 96 h were collected for MTT assay.

### Soft agar formation assay

The colony-forming ability of AGS and HGC-27 cells was measured by Soft agar formation assay. Generally, cells with or without 8 μM of WYC-209 treated were inoculated at 3000 cells/well into 6-well plates for 3 weeks, followed by methanol and crystal violet (0.5%) treatment for fixing and staining. Finally, the cell colonies were photographed and counted under a microscope (100X magnification) (Olympus Corp, Tokyo, Japan, Olympus IX73).

### Tanswell experiments

The cell migration and invasion capacities were detected using transwell assay. Generally, cells with or without 8 μM of WYC-209 treated were suspended in Dulbecco’s modified eagle’s medium (DMEM) (serum-free). The suspensions were added into the upper chamber, additionally, the upper chamber was placed into 24-well plates with 500 μl of DMEM, which contained 10% FBS, supplied. 24 h later, 4% paraformaldehyde and 0.5% crystal violet (Sigma-Aldrich, St. Louis Missouri, USA) were applied to fix and stain cells migrating across the membrane successively. Lastly, a microscope (100X magnification) was used to photograph and the migration results were obtained based on three randomly selected fields.

To detect the cell invasion ability, the PET membrane of the transwell chamber was pre-coated with the matrigel (BD, Franklin, New Jersey, USA 356,234) in advance, and the further steps were the same as those of migration experiments.

### Bioinformatics

The total RNA of HGC-27 cells was extracted after 8 μM of WYC-209 treatment for 24 h, and then RNA-seq was conducted by LC-BIO (Hangzhou, China) to screen differentially expressed genes (DEGs). The original count matrix was converted into FPKM, the gene expression of GDF1, CXCL14, GDF6, NODAL, IBSP, FGF18, FLT1, SHC3, and WNT4 from the converted FPKM matrix were extracted and conducted unpaired two-tailed Student’s t-test. R version 3.6.3 and R version 4.1.3 were utilized respectively for the heatmap and the volcano plot. According to the sequencing results, GO and KEGG enrichment analyses were performed by DAVID Bioinformatics Resources (https://david.ncifcrf.gov/). JASPAR (https://jaspar.genereg.net/) was utilized for screening the potential binding sites on the RARα promoter to WNT4.

### qRT-PCR

The total RNA of cells after treatment was refined and reversely transcribed to cDNA by a cDNA synthesis kit (Takara, Kyoto, Japan, 6215B). PCR system was generated by SYBR (Takara, Kyoto, Japan, RR420L) and the procedure was performed on the ABI-7500 system (Thermo, Waltham, MA, USA 4,351,106). Finally, the mRNA expression levels can be analyzed from the Ct value measured by the 2^−ΔΔCt^ method. β-actin worked as a control. The primer sequences are shown in Table 1.

**Table 1.** Sequences of qRT-PCR primers.

Names	F(5’-3’)	R(5’-3’)
CDF1	CTCTAGGACTGCGCGATGAG	ACTGTCCACTCAGGGCAATG
CXCL14	GGACCCAAGATCCGCTACAG	CTTCGTAGACCCTGCGCTTC
GDF6	CGGCAAGAAGTCCAGGCTAC	ATTATTGCCCGCGTCGATGT
NODAL	CCATCCCCTCTGGCGTACAT	GGTCCATCTGAAACCGCTCT
IBSP	GAACCTACAACCCCACCACA	AGCCATCGTAGCCTTGTCCT
FGF18	CAAGGGCAAGGAGACGGAAT	TGGTCACCGTCGTGTACTTG
FLT1	GGCCAGCGAGTACAAAGCTC	AGGCTCCATGTGTAGTGCTG
SHC3	TTGGAATGCTGTGATGGGCT	TGGAGGAGGCATCTTGCTTG
WNT4	GTCTTCGCCGTCTTCTCAGC	TACTGGCACTCCTCAATGGC

### Western blot (WB)

The concentration of extracted proteins was measured by the BCA method.^35^ WB experiments were operated referring to the published paper.^36^ Additionally, blocking was performed with 5% skim milk. β-actin was the loading control. After incubated with the primary antibodies (shown in Table 2) at 4 overnight and the following secondary antibodies at 26°C for 2 h, the protein bands were visualized using a chemiluminescent substrate kit (Bio-Rad, California, USA, NO.1705060) and photographed using BioDoc-it Imaging System (UVP, Upland, USA). Besides, the qualification of protein bands was conducted by Image J (National Institutes of Health, Bethesda, Maryland, USA).

**Table 2.** Information on the primary antibodies.

Name	Brand	No.	Dilution rate
β-actin	CST	#3700	1:1000
N-cadherin	CST	#4061	1:1000
E-cadherin	CST	#3195	1:1000
Vimentin	CST	#3932	1:1000
Snail	Abcam	ab216347	1:1000
Slug	Abcam	ab302780	1:1000
p-STAT3	Abcam	ab267373	1:1000
STAT3	CST	#9139	1:1000
p-AKT	CST	#4060	1:2000
AKT	CST	#4060	1:2000
p-smad2	Abcam	ab280888	1:1000
p-smad3	Abcam	ab52903	1:2000
smad2/3	CST	#8685	1:1000
β-catenin	CST	#8480	1:1000

### Transient transfection

Transfection was performed based on the pCDH-CMV-MCS-EF1-Puro vector (YouBio, Hunan, China, VT1480). To construct WNT4 overexpressed cell lines (HGC-27 and AGS), the human WNT4 CDS sequence was synthesized and cloned into the pCDH vector. Next, HGC-27 and AGS cells were transfected with WNT4 overexpressed plasmid by lipofectamine 2000 (Invitrogen, Waltham, USA 11,668–019) as the applier’s protocol.

### Construction of WNT4 stably overexpressed cell lines

The WNT4 stably overexpressed cell line was constructed using lentivirus. The WNT4-overexpressed lentivirus and the control lentivirus were obtained from GeneChem (Shanghai, China). Then, the viruses (1.0 × 10^8^ Tu·ml-1) were co-incubated with HGC-27 cells for 48 h, and puromycin (1 μg/ml, Thermo, A1113802) was used for positive screening.

### Animal experiments

The nude mice were purchased from SLAC Laboratory Animal (Shanghai, China). Eighteen 4-week-old nude mice were grouped into three (*n* = 6) and injected with the WNT4 stably over-expressed HGC-27 cells or their control cells (2 × 10^7^ cells per mouse) subcutaneously. Four weeks of feeding later, combined with WYC-209 treatment, mice were sacrificed (cervical dislocation) following the principles of animal welfare, the tumors were dissected, and the diameter and volume were measured and calculated. The animal experiments were performed following the procedures of the Guiding Principles for the Breeding and Use of Animals in China.

### Immunohistochemistry (IHC)

IHC was carried out according to the published description.^37^ In short, xenograft tumor samples were successively fixed, paraffin-embedded, and sliced thinly (about 5-μm-thick). Next, those sections were deparaffinized by xylene and alcohol, and then a 90%, 80%, and 70% gradient of alcohol was applied to remove xylene. The following steps were antigen retrieval and inactivation of endogenous peroxidase. Subsequently, the primary antibodies against β-catenin and WNT4 were applied separately to co-incubated with tumor sections at 4°C overnight, and then the secondary antibodies were added and incubated for 1 h at 26°C. Finally, after a fresh DAB solution is added, the staining results can be observed and photographed by a microscope.

### Terminal deoxynucleotidyl transferase-mediated dUTP-biotin nick end labeling (TUNEL) assay

A TUNEL assay kit (Vazyme, Nanjing, China, #A113) was obtained for the TUNEL assay. After those steps same as the previous steps of IHC, xylene was removed, then TUNEL experiments can be performed according to the protocol. DAPI was used for staining nuclei.

### Dual-luciferase reporter

The 3’UTR of human WNT4 promoter mRNA with the three potential binding sites of RARα and corresponding mutant sequence were amplified. Next, these sequences were cloned into pGL3 vector (Promega, Madison, WI, USA) for pGL3-WNT4 WT, and pGL3-WNT4 MUT1, pGL3-WNT4 MUT2, and pGL3-WNT4 MUT3. Subsequently, treated with WYC-209, the HGC-27, and AGS cells were respectively transfected with the plasmids above using lipofectamine 2000, and the relative luciferase activity was demonstrated as the ratio of firefly luciferase activity to renilla luciferase activity.

### Chromatin immunoprecipitation (ChIP)

A ChIP-Seq High Sensitivity Kit (Abcam, Cambridge, UK, ab185908) was prepared for this part. Generally, the HGC-27 cells were fixed by formaldehyde and lysed to extract the chromatin. After the chromatins were sheared, the RARα antibody was added to generate the immune complex. Next, the complexes were pelleted, and DNA was released and purified. Finally, qRT-PCR was performed for the input results. The sequence of primer was as follows: Site 3Forward: 5’-TAATAGGTGGTTTGAGGGC-3’

Reverse: 5’-TTCAGAAAGCATGCAGGTG-3’

### Statistical analysis

GraphPad Prism 6.0 was used for statistical analysis, data in this study were all shown as mean ± standard deviation (S.D.) from three repeatable experiments. Furthermore, t-test and one-way ANOVA were performed separately for difference analysis between two and multiple groups. **P<0.01, *P<0.05.

## Conclusion

In summary, our data revealed that WYC-209 showed an anti-tumor effect on GC both in vitro and in vivo by down-regulating the expression of WNT4 via RARα, making it a prospective strategy in antitumor therapy.

## Data Availability

All data generated or analyzed during this study are shown in this published article.
